# Engineered models to parse apart the metastatic cascade

**DOI:** 10.1038/s41698-019-0092-3

**Published:** 2019-08-21

**Authors:** Lauren A. Hapach, Jenna A. Mosier, Wenjun Wang, Cynthia A. Reinhart-King

**Affiliations:** 1 Nancy E. and Peter C. Meinig School of Biomedical Engineering, Cornell University, Ithaca, NY 14853 USA; 2 Department of Biomedical Engineering, Vanderbilt University, Nashville, TN 37235 USA

**Keywords:** Cancer models, Metastasis

## Abstract

While considerable progress has been made in studying genetic and cellular aspects of metastasis with in vitro cell culture and in vivo animal models, the driving mechanisms of each step of metastasis are still relatively unclear due to their complexity. Moreover, little progress has been made in understanding how cellular fitness in one step of the metastatic cascade correlates with ability to survive other subsequent steps. Engineered models incorporate tools such as tailored biomaterials and microfabrication to mimic human disease progression, which when coupled with advanced quantification methods permit comparisons to human patient samples and in vivo studies. Here, we review novel tools and techniques that have been recently developed to dissect key features of the metastatic cascade using primary patient samples and highly representative microenvironments for the purposes of advancing personalized medicine and precision oncology. Although improvements are needed to increase tractability and accessibility while faithfully simulating the in vivo microenvironment, these models are powerful experimental platforms for understanding cancer biology, furthering drug screening, and facilitating development of therapeutics.

## Introduction

**Fig. 1:**
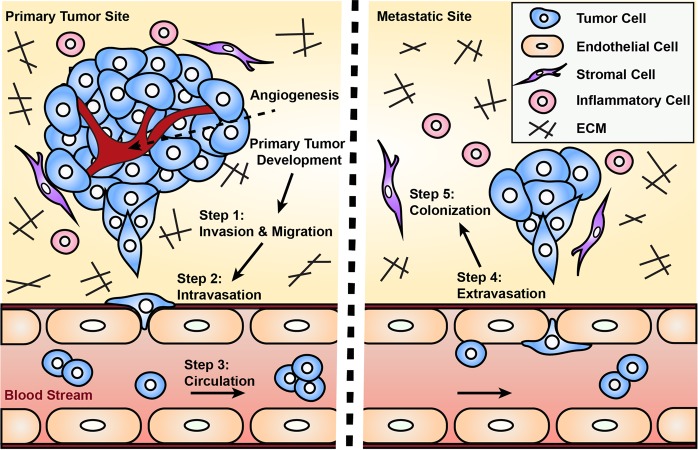
Illustrated Overview of the Metastatic Cascade. Schematic showing the essential steps in metastasis. Step 1: cancer cells invade through basement membrane and migrate through the tumor stroma; Step 2: intravasation into vasculature; Step 3: survival in the circulation is characterized by circulating tumor cells in the bloodstream undergoing shear stress and evading clearance by the immune system before reaching distant organs. After attaching to blood vessels around secondary sites, tumor cells enter; Step 4: extravasation through the endothelial barrier and Step 5: Colonization in the metastatic target organ

Metastasis is one of the leading causes of death globally.^1^ During tumor development, cancer cells acquire genetic mutations, co-opt their microenvironment, and induce angiogenic sprouting that can potentially lead to metastasis. Metastatic progression of solid tumors can be divided into five major steps: (1) invasion of the basement membrane and cell migration; (2) intravasation into the surrounding vasculature or lymphatic system; (3) survival in the circulation; (4) extravasation from vasculature to secondary tissue; and finally, (5) colonization at secondary tumor sites (Fig. 1). Each stage of metastasis imposes different, often harsh conditions and energetically taxing challenges for the cancer cells to complete. As the cascade progresses, the number of viable cancer cells which survive and successfully complete each stage decreases precipitously; however, the underlying reason for this is not clear.

Given the dynamic, multi-step nature of metastasis, and the well-documented presence of intratumor heterogeneity, certain cancer cell subpopulations may potentially perform some steps of metastasis more efficiently than others. Moreover, cooperative synergies may exist between cancer cell subpopulations such that it may not be necessary for a single subpopulation to complete the entire cascade alone.^2^ Thus, success in one aspect of metastatic fitness is not necessarily predictive of success overall. This heterogeneity complicates not only cancer studies, but more importantly, cancer diagnosis and treatment.

Several in vivo and ex ovo models have facilitated investigation of metastatic progression, yet the inherent complexity of these experiments hinders biophysical studies since effects of independent tuning of system parameters are often obscured by signaling crosstalk and homeostatic mechanisms. Most importantly, animal models are often poor predictors for human disease progression and response to treatment due to species-dependent differences and intrinsic study design limitations. These differences underscore the need for better human-like models, so much so that major federal funding agencies have released numerous calls for tissue-engineered models of cancer and metastasis in recent years. In this review, we discuss how integrating patient samples into models of the metastatic cascade advances precision oncology by faithfully reflecting the inter- and intratumor heterogeneity present during disease progression.

## Next generation cell-sourcing

Numerous cell lines have been isolated from human or murine tumors to provide homogeneous samples that possess genomic alterations consistent with their native tissue sources.^3^ However, in efforts to create more representative, heterogeneous replicas of the diseased tissue, models can now incorporate primary human tumor samples and patient-derived xenografts (PDXs).^4,5^ PDXs and primary cells obtained directly from patients are more rigorous predictors of clinical outcomes by incorporating patient-specific genomics absent from cell lines. Notably, PDXs can accurately predict clinical response to targeted cancer therapeutics.^6^ Although these must be maintained in immunocompromised mice long term and can be only used for a limited number of passages in vitro, the ability to assimilate primary human samples into engineered models is a significant advance over traditional animal models and microfabricated platforms.

With tissue banks becoming more readily available for research use, both normal and cancerous tissue samples from patients can be obtained.^7^ Tissue specimens from diagnostic surgery can be procured and manipulated to obtain primary human cells which can be immortalized for long term use by treating with human telomerase.^8^ Immortalized primary cells retain many traits from the primary tissue specimen while still undergoing multiple passages, and they can recapitulate cancer cell signaling and extracellular matrix (ECM) remodeling.^8^ The ability to incorporate primary samples into in vitro models of each metastatic stage has the potential to transform these devices into more precise and impactful predictors of clinical outcomes.

## Step 1: invasion and migration

Metastasis is initiated during invasion and migration where cancer cells penetrate the basement membrane and navigate as single cells or via collective means through the stromal microenvironment, respectively.^9^ Invasion through the basement membrane is considered the differentiating step between pre-cancerous neoplasia and malignant cancer in which increased collagen deposition, fiber thickness, and linearized fiber architecture contribute to a stiffer environment.^10,11^ Cells mechanically remodel ECM through a cycle of cell protrusion and contraction, and chemically degrade the matrix using metalloproteinases as they migrate.^12^ In addition, cancer cell contractility and matrix stiffness create a positive feedback loop causing downstream effects on cell behavior during metastatic progression.^13^ As such, accurate invasion and migration models must incorporate ECM with tunable stiffness, adjustable pore size, and measurable and/or controllable degradability.

**Fig. 2:**
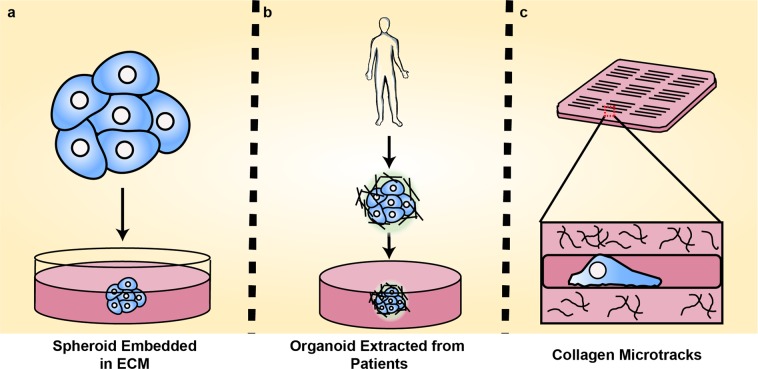
3D in vitro models of cancer cell invasion. **a** Tumor spheroids facilitate cell–cell interactions while mimicking the invasion process. **b** Organoids are self-assembled structures derived directly from human patients to recapitulate tumor environment. **c** Physiologically relevant architectures such as microtracks can be recapitulated using micropatterning and seeded with cancer cells to observe migration in these unique environments

Despite advances in tissue-engineered models*,* in vitro tumor models rarely capture the full complexity of spatiotemporal heterogeneities inherent in tumor progression due to cell culture time scales and construct size limits. The use of organoids partly overcomes these limitations by better representing genotypic and phenotypic diversity in a structured in vitro microenvironment. These structures, derived directly from human tumor tissue samples, preserve three-dimensional architecture and patient-specific phenotypes while in culture (Fig. 2a).^14,15^ Organoids capture many of the genomic variations present in solid tumors and serve as preclinical drug-screening tumor models, shown to correlate with clinical response to common cancer therapeutics.^14,15^ Long-term culture is still challenging due to insufficient nutrient and oxygen supply at the core, yet attempts at vascularization are being investigated to enhance cell maturation and model longevity.^16^

Alternatively, spheroids are cell aggregates used to study invasion and migration (Fig. 2b).^17^ Growth kinetics, heterogeneity, protein signaling, and gene expression can be captured in single or co-culture models, allowing specific characterization of the tumor microenvironment.^17–19^ In addition, spheroids can be maintained for nearly 3 weeks in ultra-low adhesion, multi-well plates, making them ideal for high-throughput screening.^17^ Reproducibility and stability make these models ideal for identifying features of the tumor microenvironment that drive metastatic cell behavior. For example, induction of hypoxia in a spheroid model was shown to be essential in eliciting the cancer stem-like cell phenotype, a major target in current cancer therapeutics.^19^ By modeling the relationship between cell behavior and the tumor microenvironment, more specific therapeutics can be developed.

Spheroid models also provide a platform to study the distinguishing factors between single cell and collective migration.^9,20^ Collectively migrating cells exhibit distinct leader-follower behavior in spheroids, with cancer cells either being led by other cancer cells or being directed by matrix fiber orientation.^20,21^ Models employing micromolding and spheroid formation in a microwell-array platform permit stromal-tumor cell interactions that can affect both the chemical and mechanical microenvironment to influence cell differentiation and migration.^22,23^

As more information is obtained on the role of cell–cell communication during invasion, engineered models must also reflect these interactions. For example, myoepithelial cells surrounding the basement membrane are thought to possess a tumor-suppressor role that can be lost during pre-cancerous neoplasia.^24^ These cells were shown to restrain and recapture cancer cells in a spheroid co-culture model. Thus, incorporation of important stromal cell types such as myoepithelial cells into invasion assays may be a promising avenue for increasing physiological relevance. It is likely that stromal-tumor cell interactions are also heterogeneous across patients, which these in vitro platforms could help define.

Cancer cells can be seeded directly into collagen matrix to investigate cell speed, direction, and morphology during migration.^25–27^ Importantly, there is now significant evidence to suggest that collagen fiber alignment is a signature of metastatic disease and can be used to predict patient outcomes.^28^ These aligned fiber architectures can be replicated in vitro through application of mechanical strain, thereby providing cells with guidance cues to direct migration.^29,30^ In addition to fiber alignment, confinement imposed by the matrix can direct cancer cell migration. Narrow tracks which confine migrating cells, polydimethylsiloxane (PDMS) posts modeling various levels of substrate rigidity, and synthesized networks of tunable porosity mimic features in tumor architecture during disease progression.^26,31,32^ To observe confined migration in a more physiologically relevant system, collagen can be micro-molded to create tracks of tunable geometries recapitulating in vivo collagen structures (Fig. 2c), offering significant advantage over stiff, PDMS-based microchannel devices.^25–27^ Collagen microtracks can provide insight into pathways driving confined migration, such as revealing the role of specific focal-adhesion proteins necessary for cell directionality, providing specific targets for clinical drug development.^27^

Perhaps one of the most significant advances of recent in vitro platforms is their ability to collect cells following invasion and migration to further analyze their physical and genetic attributes with respect to their migratory behavior.^33,34^ As just one example of the utility of this approach, cells collected post-chemotactic migration exhibited increased levels of RhoC GTPase and p38γ, which are associated with poor cancer prognosis.^34^ Although these approaches are still in early stages, they show promise in understanding the diverse molecular signatures contributing to successful migration and invasion and may lead to a better understanding of phenotypic and molecular changes cells undergo during dissemination.

## Step 2: angiogenesis and intravasation

Tumor angiogenesis refers to the formation of nascent vasculature during tumor progression, enabling delivery of nutrients and oxygen as well as the removal of waste.^35^ Newly formed tumor vasculature is immature and hyperpermeable due to lack of perivascular coverage and basement membrane, causing leakage of plasma proteins that further facilitate new vessel formation and tumor cell intravasation. Angiogenesis facilitates metastasis by enabling transport of tumor cells to distant sites via vascular and lymph systems. As such, perfusable models that enable the formation of endothelial networks aid in identification of the unique influences of angiogenesis on tumor progression.

In vitro angiogenesis assays focus primarily on cell proliferation, migration, vessel formation, and endothelial barrier integrity.^36^ Transwell models enabled the discovery of multiple pro-angiogenic factors; however, these systems lack three-dimensional cell–cell interactions and pre-existing vasculature.^36^ Recently, more advanced models have been developed to study patient-specific endothelial tubule formation and barrier function by embedding endothelial cells, including patient-derived samples, within three-dimensional hydrogels.^37–39^ However, fluid flow and growth factor gradients are absent from these models.

Since blood flow and interstitial pressure influence tumor angiogenesis, most microfluidic systems focus on recapitulating these in vitro. Models utilizing micromolding and bioprinting techniques have been used to fabricate endothelialized tissue constructs to visualize real-time endothelial cord formation during tubulogenesis.^40^ Others have incorporated a layer of human bone marrow stromal cells surrounding the channels to recapitulate perivascular-mediated barrier function.^41^ Compared with earlier models, the most recent systems endow more accurate control of growth factor gradients and fluid flow, making them ideal for patient-specific models.^42,43^ For example, a recent microfluidic angiogenic model promoted human-induced pluripotent stem cell (hiPSC) differentiation into endothelial cells which assembled into perfusable, capillary-like networks, and capturing endothelial response to different environmental biochemical and biophysical cues.^43^ Incorporation of patient-derived hiPSCs in platforms such as these lays the foundation for personalized characterization of tumor angiogenesis and endothelial cell response to cancer therapeutics. In addition, the hyaluronic hydrogel used in this platform has been shown to induce cell migration, and thus further modifications to this design could potentially be used to simultaneously investigate invasion and migration in a defined microenvironment.

Although current in vitro models contain essential features of angiogenesis, further work should be aimed at incorporating tissue-specific cell types and ECM features found at organ-specific primary tumors sites, such as recapitulating low permeability vascular beds present at the blood–brain barrier or highly permeable vascular beds of liver sinusoids.^44^ In addition, tremendous inter-tumor heterogeneity in angiogenic activity depends partly on the organ of origin and cancer subtype, due to organ-specific differences in the pro- and anti-angiogenic molecule secretion profiles of stromal cell populations.^35^ Thus, the development of more specific, personalized experimental systems will enable characterization of angiogenic behavior within different tumor types and for individual patients, leading to improvements in drug-screening models.

In addition to providing nutrients, tumor vasculature also facilitates intravasation, the process by which cells infiltrate the vasculature.^45^ The tumor microenvironment provides both chemical and physical cues to induce tumor cell intravasation. For example, stiffened ECM has been correlated with increased endothelial permeability which potentially promotes tumor cell intravasation.^46,47^ Although tumor cells secrete pro-angiogenic factors influencing the vascular phenotype, vascular cells actively regulate invasion.^48,49^ Thus, co-culture models representing the complex interactions between cancer cells, endothelium, and surrounding stroma are necessary to characterize intravasation.

Microfluidic systems allow for the incorporation of fluid flow and are amenable to real-time imaging capability. Recently, commercially available microchannel systems were used to observe intravasation events after vascular network formation.^48,50^ For example, one of these systems incorporating cancer, endothelial, and immune cells supports the role of macrophage-assisted intravasation correlating with clinical results.^48^ Thus, these platforms serve as models to examine potential immune cell involvement in intravasation.

## Step 3: survival in the circulation and attachment to the endothelium

**Fig. 3:**
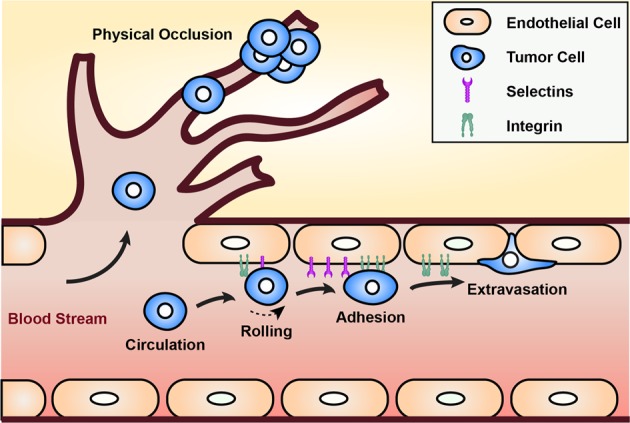
Mechanisms of cancer cell arrest in the circulation. Physical occlusion occurs when the diameter of the circulating tumor cell exceeds the diameter of the vessel it is traveling through and becomes lodged. This occurs primarily in small capillary systems. Rolling-adhesion occurs when cancer cells collide with the endothelial wall, have loose interactions with selectins (rolling), and then become more firmly attached via integrin-CAM binding (adhesion). After either of these scenarios, cancer cells can extravasate beyond the endothelium

Although few cancer cells reach the circulation, even fewer survive the hemodynamic shear forces, immune stresses, and red blood cell collisions they encounter once there.^51^ Circulating tumor cells (CTC) arrest in a vessel and extravasate through two primary mechanisms: physical occlusion and adhesion after rolling (Fig. 3). During physical occlusion, a CTC’s diameter surpasses that of the microvasculature, and the cell becomes lodged before attaching and extravasating. During rolling-adhesion, CTCs collide with the endothelium, roll via E-selectin or P-selectin binding, and arrest via intercellular adhesion molecule-1 (ICAM-1) or vascular cell adhesion molecule-1 (VCAM-1) binding. In vitro models for this process require spatiotemporal control of shear forces, tunable substrate functionalization, and real-time imaging capability.

Microfluidic and microtubing systems enabling the collection of both single and clustered CTCs from patient blood have contributed greatly to understanding cancer metastasis.^52–55^ These platforms often employ surfaces functionalized with CTC-specific adhesion proteins or antibodies to optimize adhesion dynamics for CTCs while minimizing that of leukocytes also present in whole blood.^52,53,55^ Physical entrapment under flow can also be utilized to isolate CTC clusters that have been suggested to have increased metastatic potential compared to single CTCs.^54^ Recent work has shown that single-cell encapsulation of CTCs into microdroplets can be utilized to profile enzyme secretion.^56^ In addition, single-cell RNA sequencing of human patient CTCs has been optimized to assess inter- and intra-patient heterogeneity and identify potential therapeutic targets using a microfluidic platform and barcoding technique.^57^ As the methods to capture viable CTCs become more tractable, further probing of later stages of metastasis using isolated CTCs could provide insight into the properties of these rare but crucial cells. Engineered platforms have the potential to elucidate the changes CTCs may undergo as they transition from solid tissue to the circulation, as well as determine the properties of CTCs best suited for extravasation and colonization.

Cone and plate viscometers are often used to expose cancer cells to physiological shear forces in cell culture medium or whole blood^58,59^ and study the interactions of CTCs with neutrophils, platelets, and endothelial monolayers.^60–62^ Efforts to increase throughput have led to the development of a cone viscometer platform that interfaces with standard 96-well plates to enable more streamlined testing.^63^ Although cone and plate viscometers facilitate highly controlled, reproducible exposure to shear conditions, they lack relevant vessel-like architecture and do not allow for real-time imaging during shear exposure.

Numerous commercially available, relatively inexpensive platforms are used to produce shear stresses in vitro. Motorized expulsion through a needle has been used to assess cancer cell viability and conditioning after shear stress exposure.^64,65^ Parallel plate flow chambers can be used to assess rolling-adhesion interactions between circulating cells perfused over a substrate coated with ECM, ligands, or endothelial monolayers.^58,66,67^ Others assess the rolling and adhesion of cancer cells to physiologically relevant proteins using controlled perfusion through functionalized microtubing.^68–70^

Microfluidic systems provide an immense degree of customization, with the ability to incorporate complex structures and dynamic flow patterns in perfused channels that can be coated with ECM or endothelial monolayers.^71^ More complete microfluidic models can incorporate spatially defined chemokine gradients and or organ-specific cells, such as primary lung endothelial cells or osteo-differentiated bone marrow derived stem cells.^72–74^ Although numerous microfluidic platforms mimic the vasculature, CTCs can also travel through the lymphatic system. Notably, it was observed that low shear stresses mimicking lymphatic flow induced cancer cell motility while high shear stresses mimicking arterial and venous flows inhibited cell motility in a YAP1-dependent manner, highlighting the importance of selecting physiologically relevant shear stresses since different ranges can elicit divergent cell behaviors.^75^

Tumor cell arrest during extravasation can also occur through cancer cell occlusion in capillary networks. Serial deformation and transmigration chambers in microfluidic devices have been designed to mimic constrictions in capillaries and relevant endothelial/ECM barriers that cells must bypass to transmigrate after arresting.^76^ For example, a microfluidic device with capillary-sized channels was used to show that CTC clusters isolated from patient blood can traverse these constrictions while remaining intact.^77^

Recently, self-assembled perfusable microvascular networks have been developed to investigate physical occlusion and rolling-adhesion events leading to extravasation.^78^ To stabilize self-assembled networks, co-culturing fibroblasts segregated from endothelial cells provides the necessary paracrine signaling for network stabilization, while co-seeding with pericytes regulates vessel diameter and decreases vessel permeability.^78,79^ Although self-assembled microvascular networks do not necessarily require specialized equipment, control over network formation is limited.

Three-dimensional printing of carbohydrate glass sacrificial fibers can create highly controlled, multiscale, and perfusable vascular networks.^80^ Although geared towards improving tissue engineering designs, this platform could be adapted to study extravasation in capillary networks. Live-cell lithography was developed to better control cell placement for in vitro vascular network assembly.^81^ In this system, multiple optical tweezers are used to manipulate placement of cells in three dimensions allowing the controlled addition of pericytes, smooth muscle cells, and fibroblasts outside of the vessel. Advances like these lay the foundation for systems that better recapitulate the complexity of the tumor microenvironment. As patient CTC capture platforms improve, further incorporation of these precious clinical samples into downstream assays will be crucial towards investigation of CTC performance during subsequent stages of metastasis. If assays can be streamlined and correlated with clinical data, theranostic platforms with CTCs isolated from patient blood have the potential to improve clinical outcomes.

## Step 4 and 5: extravasation and colonization

Following arrest within the vessel, cancer cells must extravasate from the vessel to colonize new sites. This process differs from intravasation, where cancer cells navigate tumor-modified stroma via chemotactic and durotactic gradients toward leaky, nascent vasculature without experiencing hemodynamic stressors; rather, during extravasation, the vasculature that is breached by cancer cells is healthier, and cancer cells actively experience fluid shear stresses due to blood flow.^82^ After extravasation, cancer cells have one final task to complete: colonization of secondary sites. This process is thought to be extremely inefficient with only a minute percentage of CTCs growing into lesions.^83^ Metastatic niches possess cell types and ECM compatible for tumor cell survival and growth,^83^ including perivascular niches, spaces around blood capillaries where cancer cells can seed. Extravasation and colonization models require tissue-specific cell types, microenvironmental cues, and vascularization. Leveraging tissue engineering advancements to model metastatic sites may be key in understanding factors driving colonization as it is possible to tailor the site to isolate roles of cells types, growth factors, and ECM architectures.

As bone metastasis occurs frequently in breast and prostate cancers and correlates with shortened patient prognoses, many models of metastatic colonization in bone have been created.^84^ Osteo-differentiated mesenchymal stem cells, mineralized hydroxyapatite-incorporated ECM, and ex vivo bone scaffolds have all been shown to elicit relevant cell behavior in in vitro bone tissue models.^85–88^ Incorporation of perfusable vascular networks in these models allows for cancer cells to be flowed though, recapitulating extravasation events at the metastatic site. Bioreactors can be used to create complex, mature tissue constructs for seeding as well as to expose seeded scaffolds to tunable, physiological compressive forces to observe colonization behavior.^89,90^

Several different models have been exploited to assess colonization in various organ systems. Decellularization of tissues including mammary fat pad, lymph node, and lungs has been used to three-dimensionally map the spatial distribution of ECM components of these tissues in health and disease.^91^ Moreover, decellularization provides a scaffold that can then be re-seeded with cancer cells to examine colonization in a simplified yet physiological setting.^92^ Decellularization proves a powerful technique to enhance and inform tissue-engineered constructs of metastatic colonization sites and assess cancer cell-ECM interactions. LiverChip® is a commercialized microfluidic model of the hepatic niche used to observe interactions between cancer cells, hepatocytes, and non-parenchymal cells.^93^ Infiltration of the brain–blood barrier has been modeled by adding cancer cells to numerous permutations of co-cultures containing endothelial cells, pericytes, glial cells, astrocytes, and cancer-associated fibroblasts.^94–98^ As lung, liver, brain, and lymph node are all extremely common metastatic sites, further work should be directed towards developing more complex, physiologically relevant in vitro models for assessment of cancer cell metastatic colonization at these distinct locations.

Currently, metastatic colonization assays are in their infancy relative to assays focusing on earlier stages. As colonization is the stage where metastasis gains its lethality and where confounding events like drug-resistance and dormancy often occur, it is promising as a key point of intervention. While much emphasis is placed on the personalized side of patient-specific cancer cells, understanding patient-specific, non-tumor cells in metastatic sites may help explain drug-resistance and dormancy.

## Full metastatic cascade models

While in vivo models can be used to study the entirety of the cascade, the complexity and timescale of metastasis limits their utility. In vitro models successfully recapitulate individual steps in metastasis, yet few encompass more than one stage in the process.

Recent work to develop a more complete metastatic platform has resulted in a microfluidic metastasis-on-a-chip model where hydrogels embedded with host tissue cells are combined with microfluidics to represent the spread of metastatic cells from primary to secondary tissue.^99^ Specifically, microfluidic chambers containing a gut tissue-like ‘source’ seeded with colon cancer cells and a liver tissue-like ‘sink’ are connected by a perfused flow channel. In this model, cancer cells can exit the gut chamber and spread to the liver chamber. This three-dimensional construct facilitates drug-screening and visualization of metastasis, though it still lacks important features like endothelial barrier function, intravasation, and extravasation. Despite limitations, it marks one of the first steps toward an in vitro model distilling the key components representing the diverse microenvironments cancer cells encounter during metastasis.

Although there are still limitations hindering in vitro recapitulation of the full metastatic cascade, approaches that stitch together multiple sequential steps into a single assay have fewer impediments. Reductionist models that incorporate a primary tumor site and a metastatic niche site separated by ECM serve as a simplified approach to assessing metastatic potential.^100^ However, metastasis is a dynamic, multi-step process and by simplifying models to exclude parts of the cascade, we are only gaining insight as to how well cancer cells perform specific steps out of context. Thus, it is critical that more complete models be developed so that metastasis can be observed in the correct series of events.

## Enhancing individualized cancer therapeutics

**Fig. 4:**
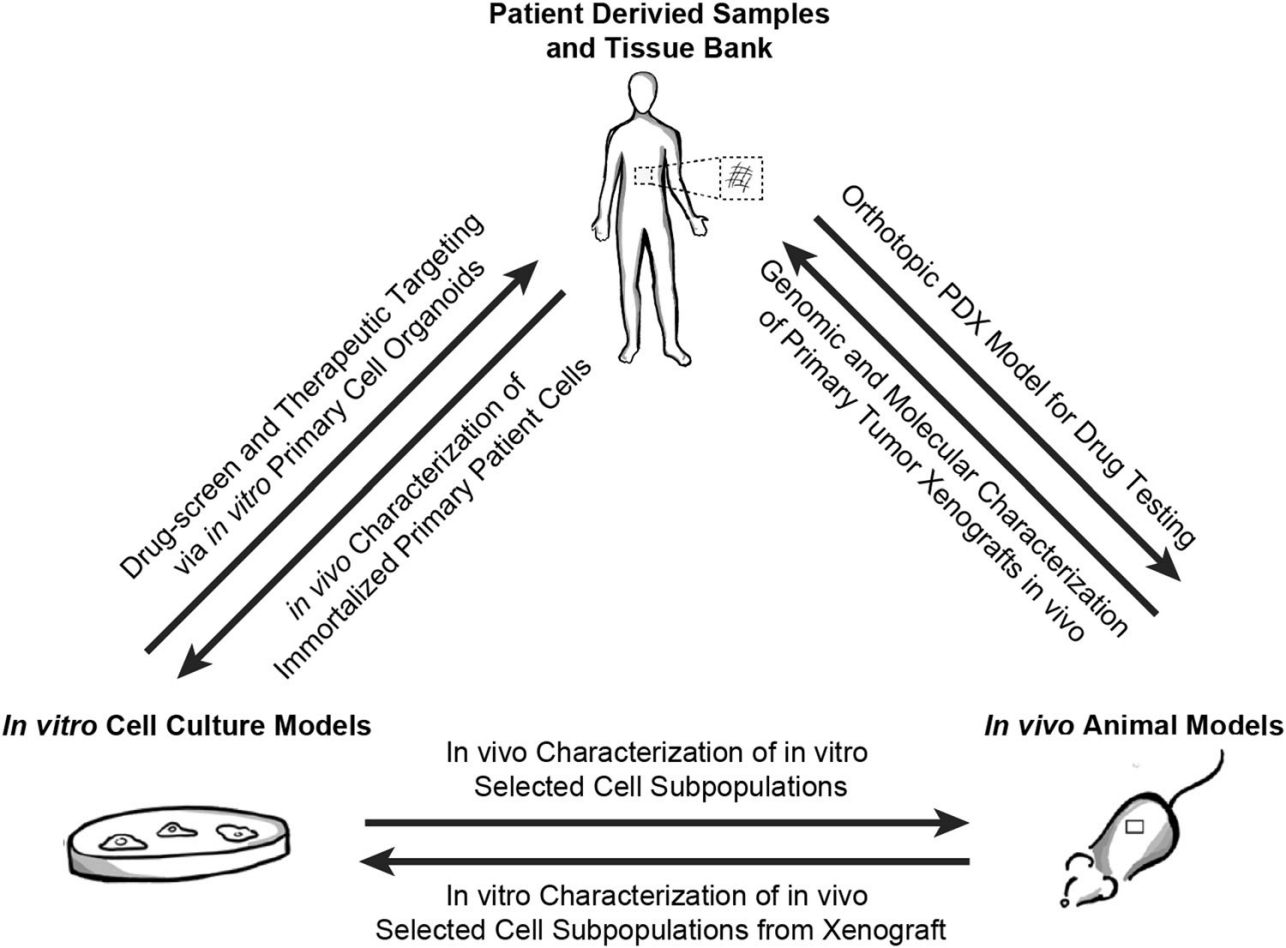
Precision oncology approaches to cancer metastasis studies illustrates the flow of samples and information gained from the different types of models used to study the metastatic cascade. Patient-derived tissue samples can be used directly in in vivo models or for characterization in in vitro platforms. In vitro models incorporating primary samples can be used to inform drug-development or characterize subpopulations of cells to be used in in vivo models. In vivo tissues can be extracted for characterization in in vitro platforms to better inform future therapeutics for human patients

Engineered in vitro models have greatly expanded our understanding of cancer metastasis. Incorporation of primary cells and tissue within metastatic models provides more physiologically relevant and clinically applicable findings that often correlate with patient outcomes, aiding in drug-screening and personalized medicine to advance precision oncology. As tissue banks become more common and access to primary human samples increases, metastatic models are moving towards more faithful representations of native in vivo cell interactions and behaviors (Fig. 4).

With the addition of primary patient blood or tumor tissue samples into established in vitro models of the metastatic cascade, personalized characterization of metastatic cancer cell behavior is gaining tractability. Further, coupling these patient-specific assays with high-throughput drug-screening approaches could aid in optimizing patient treatment plans as well as facilitate drug discovery. For example, patient-derived organoids can serve as effective preclinical models for rapidly assessing therapeutics, shown to exhibit similar responses to chemotherapeutic drugs such as topotecan and melphalan consistent with clinical outcomes.^14,15^

As microfabrication techniques and biomaterials advance, models are gaining the ability to recapitulate multiple tissue-specific microenvironments connected in a physiologically relevant manner as pioneered primarily for pharmaceutical toxicity studies. Adaptation of these systems to simulate key elements of multiple metastatic stages in sequence could provide novel insight. In addition, more comprehensive models such as the metastasis-on-a-chip model that elegantly incorporates multiple steps still lack essential components in their design, such as endothelial barriers to study intravasation and extravasation effects.^99^ Moving forward on the path towards personalized cancer theranostics, ameliorations to existing in vitro models, including the addition of patient-derived samples and integration of multiple steps of the metastatic cascade into one platform, will be essential.

### Reporting summary

Further information on research design is available in the Nature Research Reporting Summary linked to this article.

## Supplementary information

Reporting Summary

## Supplementary information

**Supplementary Information** accompanies the paper on the *npj Precision Oncology* website (10.1038/s41698-019-0092-3).
